# Anti-Proliferative Effect of Radiotherapy and Implication of Immunotherapy in Anaplastic Thyroid Cancer Cells

**DOI:** 10.3390/life13061397

**Published:** 2023-06-15

**Authors:** Sabine Wächter, Silvia Roth, Norman Gercke, Ulrike Schötz, Ekkehard Dikomey, Rita Engenhart-Cabillic, Elisabeth Maurer, Detlef K. Bartsch, Pietro Di Fazio

**Affiliations:** 1 Department of Visceral, Thoracic and Vascular Surgery, Philipps University Marburg, Baldigerstrasse, 35043 Marburg, Germany; seckhard@med.uni-marburg.de (S.W.); rothsi@med.uni-marburg.de (S.R.); gercken@staff.uni-marburg.de (N.G.); maurere@med.uni-marburg.de (E.M.); bartsch@med.uni-marburg.de (D.K.B.); 2 Department of Radiotherapy and Radio Oncology, Philipps University Marburg, Baldigerstrasse, 35043 Marburg, Germany; ulrike.schoetz@staff.uni-marburg.de (U.S.); rita.engenhart-cabillic@uk-gm.de (R.E.-C.); 3 Laboratory of Radiobiology & Experimental Radiooncology, University Medical Center Hamburg Eppendorf, Martinistrasse 52, 20246 Hamburg, Germany; dikomey@uke.de

**Keywords:** anaplastic thyroid cancer, radiotherapy, checkpoint inhibitor, apoptosis, cell death

## Abstract

Radiotherapy and immunotherapy have shown promising efficacy for the treatment of solid malignancies. Here, we aim to clarify the potential of a combined application of radiotherapy and programmed cell death-ligand 1 (PD-L1) monoclonal antibody atezolizumab in primary anaplastic thyroid cancer (ATC) cells. The radiation caused a significant reduction in cell proliferation, measured by luminescence, and of the number of colonies. The addition of atezolizumab caused a further reduction in cell proliferation of the irradiated ATC cells. However, the combined treatment did not cause either the exposure of the phosphatidylserine or the necrosis, assessed by luminescence/fluorescence. Additionally, a reduction in both uncleaved and cleaved forms of caspases 8 and 3 proteins was detectable in radiated cells. The DNA damage evidenced the over-expression of TP53, CDKN1A and CDKN1B transcripts detected by RT-qPCR and the increase in the protein level of P-γH2AX and the DNA repair deputed kinases. PD-L1 protein level increased in ATC cells after radiation. Radiotherapy caused the reduction in cell viability and an increase of PD-L1-expression, but not apoptotic cell death in ATC cells. The further combination with the immunotherapeutic atezolizumab could increase the efficacy of radiotherapy in terms of reduction in cell proliferation. Further analysis of the involvement of alternative cell death mechanisms is necessary to clarify their cell demise mechanism of action. Their efficacy represents a promising therapy for patients affected by ATC.

## 1. Introduction

Anaplastic (undifferentiated) thyroid carcinoma (ATC) is one of the rarest carcinomas of the thyroid gland and has a severely poor prognosis with a median overall survival (OS) after an initial diagnosis of 3–6 months [1,2,3,4]. In addition to the established therapy options consisting of surgery, chemotherapy and radiotherapy, targeted treatment options based on multi-kinase inhibitors (mKI) in combination with immune checkpoint inhibitors (ICI) have shown promising results for the treatment of patients affected by ATC [5,6,7]. Preclinical studies have shown that approximately 65–90% of ATC cells are positive for the expression of the programmed cell death-ligand 1 (PD-L1) and have a high frequency of PD-L1 amplifications, leading to the conclusion that ATC may provide a good target for PD-L1 inhibitors [8,9,10]. In this context, the programmed cell death-1 (PD-1)-inhibitor pembrolizumab has gained importance for the treatment of ATC and has shown promising results. Unfortunately, some tumors do not respond to or show progressive disease following PD-L1 inhibitor therapy [11,12]. Recent studies have revealed that ionizing radiation (IR) can upregulate PD-L1 expression and synergically augment antitumor immunity when applied in combination with ICI [13,14]. Sato et al. demonstrated that the expression of PD-L1 was upregulated in response to DNA double-strand breaks (DSB) in cells of solid carcinomas [15]. The DSB is the most lethal lesion, which can be induced by IR and trigger DNA damage response (DDR) and associated proteins such as H2A histone family member X (γH2AX) and signaling pathways including ataxia-telangiectasia mutated (ATM), ATM- and Rad3-Related (ATR) and DNA dependent protein kinase (DNA-PKcs) [16,17]. The crosslink between DDR signaling and immune checkpoints in ATC has not been examined yet. This study elucidated the correlation between IR-induced DSB and the expression of PD-L1 in ATC to broaden the understanding of the molecular mechanism underlying PD-L1 regulation and to highlight the combination therapy of external beam radiation therapy (EBRT) and immunotherapy with atezolizumab as a promising therapeutic option for patients suffering from ATC. Furthermore, DNA damages lead to the activation of p53, which activates the intrinsic (mitochondrial-mediated) and extrinsic (death receptor-mediated) apoptotic pathway [18,19]. Induction of apoptosis by IR is a known strategy for killing malignant cells, but no data elucidate this molecular mechanism in ATC cells.

Therefore, this study focused to investigate the cytotoxicity and the induction of apoptosis mediated by radiotherapy (RT) and by the further administration of the monoclonal antibody against PD-L1 atezolizumab in ATC. The goal was to determine the long-term effects of such treatment in terms of DSB reparatory machinery activation and apoptosis in ATC cells and the potentially lethal effect of the administration of atezolizumab.

## 2. Materials and Methods

### 2.1. Cell Lines

The C643 human anaplastic thyroid carcinoma cells, kindly provided by Prof. A. Zielke (Diakonie-Klinikum Stuttgart; Stuttgart, Germany) and human thyroid follicular epithelial cells Nthy-ori-3-1 (MERCK-Sigma-Aldrich Chemie GmbH, Schnelldorf, Germany) were grown in RPMI 1640 (Gibco^®^ by Life Technologies TM, Carlsbad, CA, USA) supplemented with 10% of fetal bovine serum (Gibco) and 10 U/mL penicillin and 100 µg/mL streptomycin (Gibco) under standard conditions (37 °C, 5% CO_2_) and routinely tested for Mycoplasma contamination.

### 2.2. Preparation of Patient-Derived Human Tumor Tissue (PDTT)

The preparation of patient-derived human tumor tissue (PDTT) from four patients who underwent surgery due to ATC was performed as previously described [20]. The tumor tissue resected from 4 patients who underwent surgery was immediately collected in sterile Phosphate-Buffer saline (PBS) without Ca^2+^ and Mg^+^ (L1825 Biochrom, Berlin, Germany). The tissue was washed (3) with sterile PBS in order to remove any tissue debris and blood. Afterward, the tissue was cut into small pieces with a sterile scalpel (Feather, Osaka, Japan). The small pieces were rinsed through a cell strainer (352350 BD Labware, Franklin Lakes, NJ, USA) and washed with Roswell Park Memorial Institute 1640 (RPMI1640) Medium (FG1215 Biochrom, Berlin, Germany). The cell suspension was centrifuged at 1500 rpm for 8 min at room temperature. The pellet was suspended with complete growth medium RPMI 1640 (Biochrom) supplemented with 10% fetal bovine serum (FBS, Biochrom) and 10 U/mL penicillin and 100 g/mL streptomycin (Biochrom) and the suspension was pipetted in a cell culture 6-well plate (83.3920 Sarstedt, Nümbrecht, Germany). After 2 h, the adherence of the cells was checked. The medium was changed regularly every second day. The cells were then trypsinized and transferred into 25 cm^2^ flasks. All PDTT were grown in RPMI 1640 (Biochrom) supplemented with 10% fetal bovine serum (FBS, Biochrom) and 10 U/mL penicillin and 100 g/mL streptomycin (Biochrom) under standard conditions (37 °C, 5% CO_2_). They were routinely tested for Mycoplasma contamination.

### 2.3. Irradiation

Cells were irradiated with the XRad 320iX irradiation cabinet (Precision X-ray Inc., Denver, CO, USA), 8 mA and 320 kV at a dose rate of 1.0 Gy/min. A filter with 0.5 mm Al/0.5 mm Cu was used.

### 2.4. Compounds Tested

Atezolizumab (A2004) was purchased by Selleck Chemicals Llc (Houston, TX, USA). The compound was dissolved in dimethyl sulfoxide (DMSO) (WAK Chemicals; Steinbach, Germany) and stored at −20 °C/−80 °C.

### 2.5. Measurement of Cell Viability

The C643, PDTT of four patients and Nthy-ori-3-1 cells viability was monitored by the use of the RealTime Glo^®^ kit (G9711) purchased from Promega (Mannheim, Germany) by following the instructions of the manufacturer. 500 cells were seeded in a 96-well plate. The next day, the cells were irradiated with 4 and 6 Gy. The measurement was acquired by FLUOstar OPTIMA (BMG LABTECH, Ortenberg, Germany) plate reader for up to 10 days. The stability of the reagents has been preliminarily tested and it has been previously published [21]. The results obtained from biological triplicates were analyzed by Excel 2016.

### 2.6. Colony Formation Assay

The C643, four PDTT and Nthy-ori-3-1 cells were seeded at a density of 500,000 in 75 cm^2^ flasks. The next day, the cells were irradiated with 4 and 6 Gy. 24 h after radiation, the cells were suspended by trypsinization, counted and seeded in 6-well plates at different densities, based on the preliminary test of their best colony forming density. The range for all cells was between 200 and 1200 per well. The colony counting was performed 14 days after seeding. The cells were fixed and stained for colony counting (≥50 cells) with a 1 g/L crystal violet (MERCK-Sigma-Aldrich) in formaldehyde (MERCK-Sigma-Aldrich) (10% diluted in PBS) solution for 30 min. After washing with PBS, the presence of colonies was determined by stable violet staining.

### 2.7. Quantitative RT-PCR

The C643, Nthy-ori-3-1 and primary anaplastic thyroid cancer cells were irradiated with 4 and 6 Gy. Total RNA was collected, 1 day and 7 days after the irradiation, by using the RNeasy Mini Kit (74106, QIAGEN, Hilden, Germany) according to the manufacturer’s protocol. The cDNA was obtained by using iScript TM cDNA Synthesis Kit (170-8891, BIORAD, Hercules, CA, USA) on FlexCycler (Analytik Jena AG, Jena, Deutschland). Qiagen primers for human CDKN1A (QT00062090), CDKN1B (QT00998445), TP53 (QT00060235) GAPDH (QT01192646) were mixed with GoTaq^®^ qPCR Master Mix (Promega, Madison, WI, USA) on RT-qPCR thermocycler CFX96TM Real-Time System (Bio-Rad Laboratories, Hercules, CA, USA). Results were analyzed with the Bio-Rad CFX-Manager (Bio-Rad Laboratories) and normalized to GAPDH and to untreated cells’ mRNA content for each sample. Raw data were further processed with Rest2009 (relative Expression Software Tool V.2.0.13. Qiagen).

### 2.8. Measurement of Apoptosis/Necrosis

The detection of apoptosis/necrosis was performed by luminescence/fluorescence after the administration of the RealTime-Glo™ Annexin V Apoptosis and Necrosis Assay (JA1011, Promega). A total of 1000 cells of Patient 1, Patient 2, Patient 3, Patient 4, C643 and Nthy-ori-3-1 were seeded in 96-well plates. After 24 h, the cells were irradiated with 4 and 6 Gy. Seven days after radiation, 500 ng/mL of atezolizumab was administered to the cells. The measurement was acquired by FLUOstar OPTIMA (BMG LABTECH, Ortenberg, Germany) plate reader for up to 72 h. The data obtained from biological triplicates were analyzed by Excel 2016 (Microsoft).

### 2.9. Western Blot Analysis

The C643, Nthy-ori-3-1 and primary anaplastic thyroid cancer cells were irradiated with 4 and 6 Gy. Whole-cell lysates were isolated in Jie’s Buffer (10 mM NaCl, 0.5% NonidetP40, 20 mM Tris-HCL pH7.4, 5 mM MgCl_2_, 1 mM PMSF, Complete Protease Inhibitor and Phosphatase Inhibitor (Roche, Basel, Switzerland)) one day and seven days after irradiation. The proteins were separated through SDS-Page (NP0342, Life Technologies, Carlsbad, CA, USA) and transferred to nitrocellulose membranes (10600009, GE Healthcare Life science, Chicago, IL, USA) by semi-dry-blotting with Trans-Blot^®^TurboTM Transfer System (Bio-Rad Laboratories). The membranes were further sliced according to the required molecular weight of the proteins of interest, blocked in 4% BSA (23208, Thermo Fisher Scientific, Waltham, MA, USA) in TBS-Tween20 (0.5%) and incubated with primary antibodies against PD-L1 (ab205921, AbCam, Cambridge, UK), P-γH2AX (#9718T, Cell Signaling, Cambridge, UK), ATM, P-ATM, ATR, P-ATR, DNA-PKcs, P-DNA-PKcs (DNA Damage Kinases Panel, ab103970), Caspase 8 (ALX-804-242, Enzo Life Sciences GmbH, Lörrach Germany), Caspase 3 (NB100-56708, Novus Biologicals, Abingdon, UK) and β-actin (A5441 SIGMA-ALDRICH, St. Louis, MO, USA). Bound primary antibodies were detected by secondary horseradish-labeled goat anti-rabbit (A0545, SIGMA-ALDRICH) and goat anti-mouse (A9917, SIGMA-ALDRICH) antibodies and SuperSignal West Pico Chemiluminescent Substrate (Thermo Fisher Scientific, Waltham, MA, USA). The immuno-detection was quantified using Fusion image capture (VILBER LOURMAT Deutschland GmbH, Eberhardzell, Germany) and Bio-1D Analysis System (VILBER LORUMAT Deutschland GmbH).

### 2.10. Statistical Analysis

If not stated otherwise, all experiments were performed in triplicates and repeated at least three times. Data were collected using Excel (Microsoft Office). Significance was calculated using the *t*-test for paired samples. *p* < 0.05 was considered significant (*). The analysis of the survival and its graph were made by using GraphPad Prism version 9.0 (GraphPad Software Inc., La Jolla, CA, USA).

### 2.11. Ethical Approval

The study was conducted according to the guidelines of the Declaration of Helsinki and approved by the Institutional Ethics Committee of the University Hospital of Marburg (No.123/19). Informed consent was obtained from all subjects involved in the study.

## 3. Results

### 3.1. Effects of Radio- and Immunotherapy on the Cell Proliferation of ATC Cells

**Figure 1:**
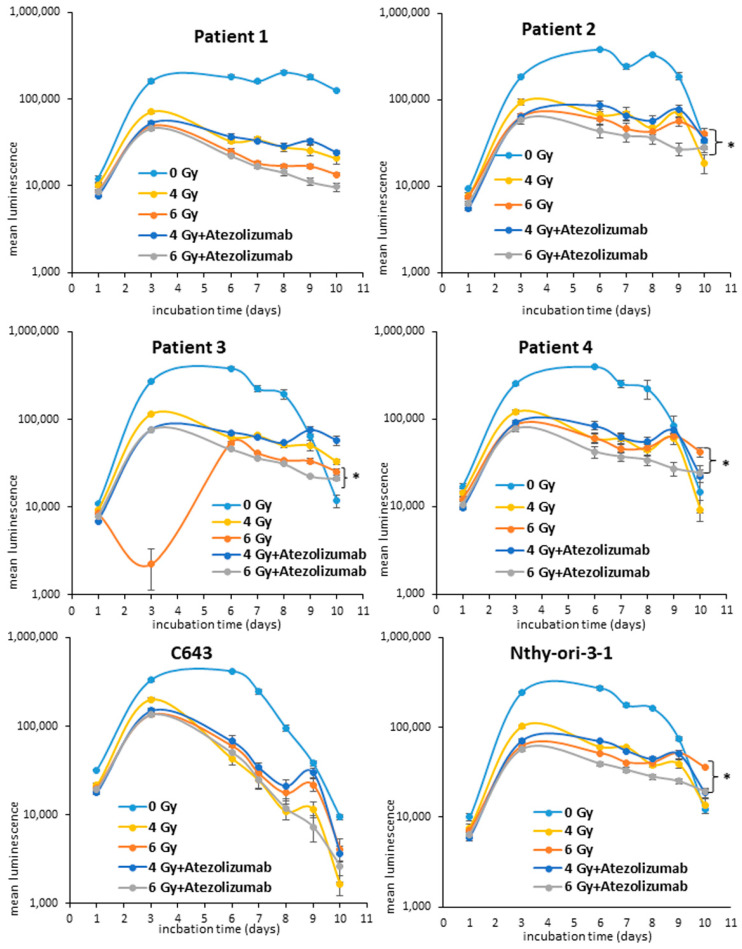
**Analysis of cell viability of ATC cells after radiotherapy**. Patient 1, Patient 2, Patient 3, Patient 4, C643 and Nthy-ori-3-1 cells were initially exposed to radiation (4–6 Gy). 500 ng/mL of atezolizumab was administered 7 days post radiation. The cell viability (log10) was measured by luminescence every 24/48 h for 10 days. Shown are means ± SEM of three independent experiments performed in triplicates. * *p* < 0.05 of radiation treated cells vs. radiation and atezolizumab treated cells.

Previous radiotherapy studies have highlighted that ATC cells are sensitive to low irradiation dosage already [22]. The cells included in this study have been preliminary irradiated, thus showing their sensitivity to 4 and 6 Gy. For this reason, this irradiation intensity has been adopted for all the following experiments. The radiotherapy with 4 and 6 Gy caused a reduction in the cell proliferation of all cells included in the study (Figure 1). This effect was observed for the following 10 days after irradiation. Furthermore, it could be observed that the 72 h of administration of 500 ng/mL of atezolizumab (day 7 after irradiation), a monoclonal antibody binding PD-L1, to 6 Gy irradiated cells, affected significantly (* *p* < 0.05) the cell proliferation of Patient 2, Patient 3, Patient 4 and Nthy-ori-3-1 cells between day 7 and day 10 (Figure 1). The administration of atezolizumab to 4 Gy irradiated cells caused no significant reduction in cell viability in comparison to 4 Gy irradiated cells (Figure 1). The administration of atezolizumab was performed in respect of the clinical standard protocol, which indicates the administration of such antibody 7 days after the patient has been irradiated. The long-term cultivation of the untreated cells (0 Gy) caused the enrichment of well-confluence after almost eight days thus leading to a block of cell proliferation with a consequent reduction in the cell viability (Figure 1).

### 3.2. Apoptotic Effects of Radio- and Immunotherapy in ATC Cells

**Figure 2:**
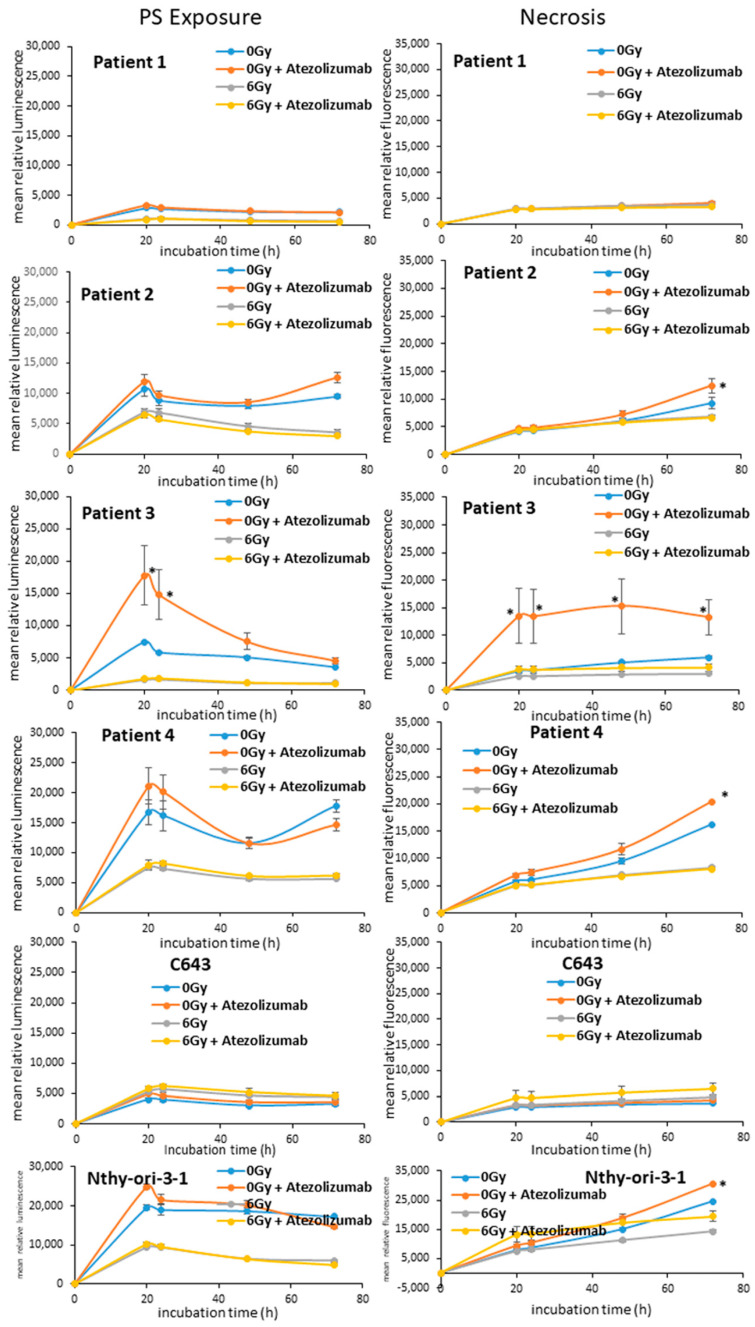
**Analysis of and apoptosis/necrosis of ATC cells after radiotherapy.** Patient 1, Patient 2, Patient 3, Patient 4, C643 and Nthy-ori-3-1 cells were initially exposed to radiation (6 Gy). 500 ng/mL of atezolizumab was administered 7 days post radiation. Phosphatidil-serine exposure (luminescence) and DNA damage/necrosis (fluorescence) was measured every 24 h for up to 72 h. Shown are means ± SEM of three independent experiments performed in triplicates. * *p* < 0.05 of radiation/atezolizumab treated cells vs. untreated (0 Gy) cells.

The reduction in cell proliferation can be attributed to the induction of cell death mechanisms such as apoptosis. In order to prove if the irradiated ATC cells are going to die by apoptosis, they were irradiated with 6 Gy and the exposure of phosphatidylserine (PS) (luminescence) and the DNA damage (fluorescence) were monitored for up to 72 h after the addition of 500 ng/mL of atezolizumab 7 days after irradiation. The administration of atezolizumab was performed in respect of the clinical standard protocol, which indicates the administration of such antibody 7 days after the patient has been irradiated. As shown in Figure 2, the radiotherapy alone and after the addition of atezolizumab did not cause either exposure of PS or necrosis. Instead, the solo administration of 500 ng/mL of atezolizumab caused an increase in luminescence/PS exposure and fluorescence/necrosis in Patient 2, Patient 3, Patient 4 and Nthy-ori-3-1 cells. The C643 and Patient 1 cells showed neither apoptosis nor necrosis after radiotherapy and immune therapy.

### 3.3. Analysis of the Guardian of the Cell Destiny after Radiotherapy

**Figure 3:**
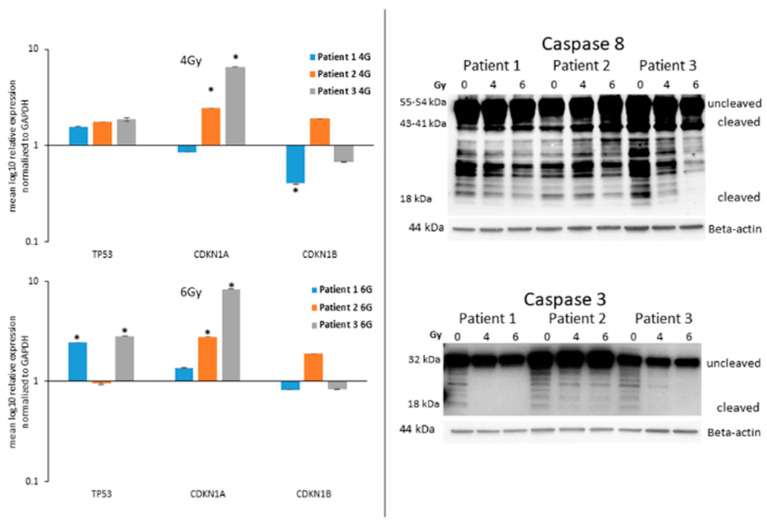
**Detection of TP53, CDKN1A and CDKN1B transcripts and the protein level of caspase 8 and caspase 3 after radiation.** (**Left panels**) Detection of the transcript level of TP53, CDKN1A and CDKN1B in ATC cells, seven days after irradiation (4 Gy and 6 Gy). Shown are means ± SEM of three independent experiments performed in triplicates. * *p* < 0.05 of radiation treated cells vs. untreated cells. (**Right panels**) Detection of the protein level (uncleaved and cleaved) of caspase 8 and caspase 3 in ATC cells, seven days after irradiation (4 Gy and 6 Gy). Beta-actin was detected as equal loading control.

All cells were monitored for the expression of the transcripts for TP53, CDKN1A and CDKN1B after irradiation with 4 and 6 Gy. Interestingly, 7 days after 4 Gy radiotherapy, a significant (* *p* < 0.05) over-expression of CDKN1A was observed in Patient 2, Patient 3 (Figure 3), Nthy-ori-3-1 and C643 (Supplementary Figure S1) cells in comparison to untreated cells. Patient 1 cells evidenced a significant down-regulation of CDKN1B (Figure 3). Only the C643 highlighted a significant over-expression of TP53 (Supplementary Figure S1). Similarly, 6 Gy radiotherapy caused the significant (* *p* < 0.05) over-expression of CDKN1A in Patient 2, Patient 3 (Figure 3), Nthy-ori-3-1 and C643 (Supplementary Figure S1) cells in comparison to untreated cells. The TP53 was over-expressed in Patient 1, Patient 3 (Figure 3) and Nthy-ori-3-1 (Supplementary Figure S1) cells. The CDKN1B transcript level was stable in all irradiated cells in comparison to untreated cells.

### 3.4. Expression of Caspases in ATC Cells after Radiotherapy

The caspases are the key player in the terminal phase of apoptotic cell death. They represent the gold standard markers for the detection of apoptosis and the final degradation of the cellular content [23]. The protein level of the initiator caspase 8 and the executioner caspase 3 was monitored in ATC cells after radiation with 4 and 6 Gy. Patient 1, Patient 2 and Patient 3 cells highlighted, 7 days after irradiation, a stable protein level of the uncleaved (55 kDa) and cleaved (44 kDa) forms of caspase 8 after radiation. Interestingly, the protein level of the smallest cleaved active form (18 kDa) was significantly downregulated and even not detectable in the 6 Gy irradiated Patient 3 cells (Figure 3). The uncleaved (32 kDa) caspase 3 was stable or slightly downregulated after irradiation of Patient 1, Patient 2 and Patient 3 cells. The cleaved form (18 kDa) was not detectable in all cells after 4 and 6 Gy irradiation. Similar results were observed in C643, Patient 4 and Nthy-ori-3-1 cells too (Supplementary Figure S1). These results excluded any involvement of the caspases in the mechanisms activated in irradiated ATC cells.

### 3.5. Radiated ATC Cells Lose Their Ability to Build up Colonies

**Figure 4:**
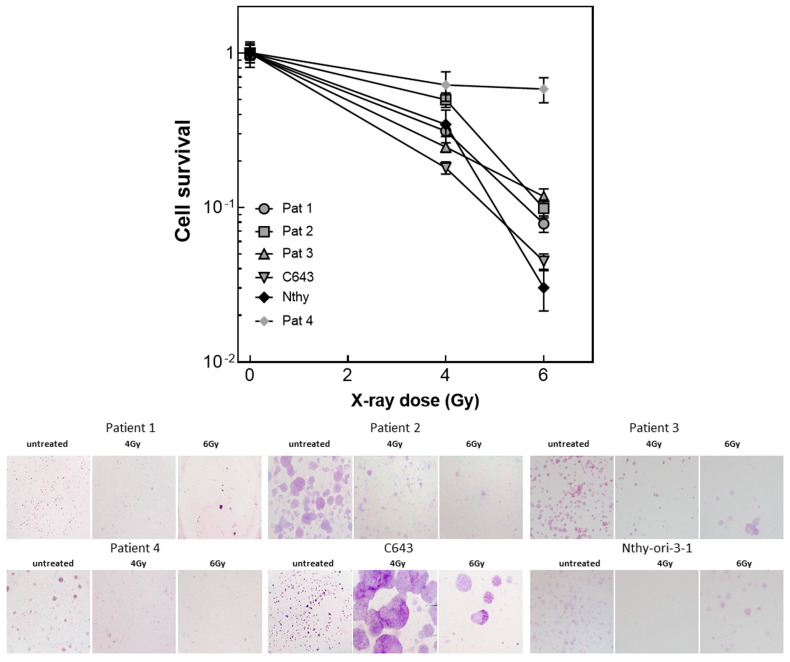
**Colony assay of ATC cells after radiation.** Patient 1, Patient 2, Patient 3, Patient 4 and Nthy-ori-3-1 cells were seeded 24 h after irradiation (4 and 6 Gy) at different density. The colonies were counted 14 days after seeding. Shown is the cell survival calculated by GraphPad Prism and the photographs of the colonies stained with crystal violet. The colonies shown in the photographs were generated by a starting suspension of 800 cells.

All cells included in the study, Patient 1, Patient 2, Patient 3, Patient 4, C643 and Nthy-ori-3-1 (Figure 4) were first seeded in 75 cm^2^ flasks and irradiated with 4 and 6 Gy. After 24 h, the cells were seeded in 6-well plates at different cell densities (200–1200 cells/well). Patient 1, Patient 2, Patient 3, Patient 4, C643 and NThy-ori-3-1 cells (Figure 4) evidenced a significant reduction in the number of colonies after 14 days of cultivation. The analysis of the cell survival demonstrated the sensitivity of almost all cells to irradiation. Only Patient 4 cell survival was not significantly affected by irradiation.

### 3.6. ATC Cell DNA Damage/Repair Machinery after Radiotherapy

**Figure 5:**
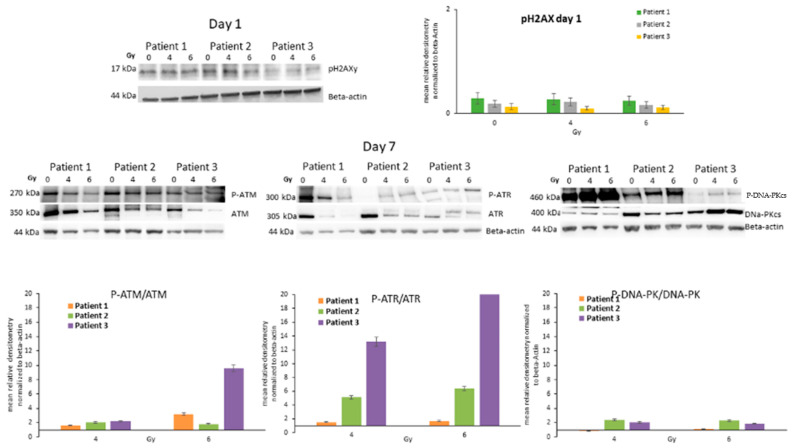
**Analysis of the players of the DNA Strand Breakage Repair Machinery.** (**Upper panels**) Detection of protein level of P-γH2AX, (**lower panels**) ATM/P-ATM, ATR/P-ATR and DNA-PKcs/P-DNA-PKcs in ATC cells, seven days after irradiation (4 Gy and 6 Gy). Bars are means ± SEM of the relative densitometry (P-ATM/ATM, P-ATR/ATR and P-DNA-PKcs/DNA-PKcs) of three independent experiments performed in triplicates.

In order to check the DNA damage caused by the radiotherapy, the protein level of the phosphorylated active form of γH2AX was detected in all cells, 24 h after irradiation with 4 and 6 Gy. As shown in Figure 5, Patient 1, Patient 2 and Patient 3 cells expressed a stable protein level of P-γH2AX, as well as Patient 4 cells (Supplementary Figure S2). Nthy-ori-3-1 cells evidenced a significant up-regulation after 4 Gy irradiation. Instead, C643 cells were characterized by a significant down-regulation of P-γH2AX protein level (Supplementary Figure S2).

Furthermore, the protein level of ATM, ATR, DNA-PK and their active phosphorylated forms were detected in all ATC cells 7 days after exposure to 4 and 6 Gy. Patient 1, Patient 2 and Patient 3 cells showed an increased protein level of the active phosphorylated form of the DNA repair machinery deputed kinases P-ATM, P-ATR and P-DNA-PKcs 7 days after irradiation of 4 and 6 Gy (Figure 5). Similar results were observed in Patient 4, C643 and Nthy-ori-3-1 cells too (Supplementary Figure S2).

Thus, highlights that the irradiation has caused DNA double-strand damage and the repair mechanism has been activated.

### 3.7. Modulation of PD-L1 after Irradiation

**Figure 6:**
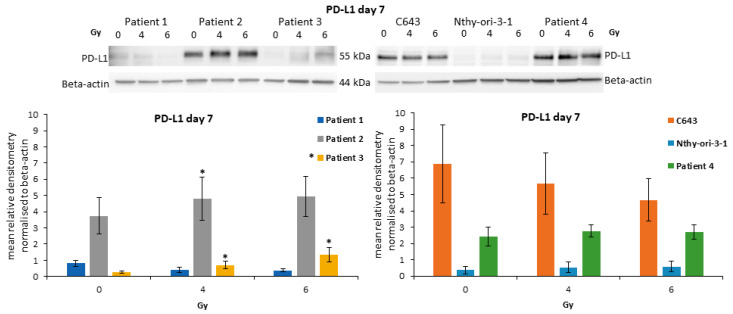
**Expression of PD-L1 in ATC cells after irradiation.** Protein level of PD-L1 in irradiated ATC cells and Nthy-ori-3-1 follicular epithelial thyroid cells. Densitometry Graph (**lower graphs**): Shown are means ± SEM of three independent experiments performed in triplicates. * *p* < 0.05 of untreated cells vs. radiation treated cells.

The protein level of PD-L1 was detected in Patient 1, Patient 2, Patient 3 as well as in C643, Nthy-ori-3-1 and Patient 4 seven days after irradiation with 4 and 6 Gy (Figure 6). Patient 2 and Patient 3 cells showed a significant up-regulation of PD-L1 after 4 and 6 Gy. Instead, Patient 1, C643, N-thy-ori-3-1 and Patient 4 cells showed a stable expression or a slight downregulation. Thus, highlighting that the modulation of PD-L1 could not represent a main process occurring in all ATC cells but a personalized effect.

## 4. Discussion

Despite its low incidence, ATC is characterized by a poor prognosis. This study highlighted the potential of photon-based radiotherapy combined with the immune checkpoint inhibitor of PD-L1 atezolizumab to inhibit ATC cell proliferation. Interestingly, the potential of radiotherapy has been already observed in ATC [24]. In particular, EBRT has shown an improvement in overall survival and a reduction in metastasis after incomplete resection of ATC [25,26] and in combination with chemotherapy [27]. However, its possible combination with the checkpoint PD-L1 inhibitor atezolizumab for the treatment of ATC has not been highlighted yet. Such a combination could further improve the patient survival and further inhibit the metastasis by targeting several pathways at once and, in particular, those tumors which highlighted an over-expression of PD-L1 and a deficient mismatch repair machinery [28,29]. The current study evidenced an effective reduction in cell viability of primary ATC cells after irradiation and administration of atezolizumab. Additionally, similar effects have been observed in commercially available ATC cells C643 and human thyroid follicular cells Nthy-ori-3-1 cells, which have been included in the study as reference cell lines. A further significant reduction caused by the additional administration of atezolizumab to irradiated cells was observed in cancer cells derived from resected tumor tissue of patients operated at the Marburg University Hospital. Additionally, the combined radiotherapy immunotherapy affected human epithelial follicular cells as well. The block of cell proliferation could be further associated with the over-expression of TP53, CDKN1A and CDKN1B transcripts. These genes encode proteins responsible for the blocking of the cell cycle and further senescence or cell death [30,31,32]. The ATC cells, which have been irradiated with 6 Gy, highlighted seven 7 days after irradiation, a significant over-expression of the TP53 and CDKN1A transcripts. Thus, this represents the first evidence of DNA damage response and the further explanation of the block of cell proliferation.

Interestingly, the reduction in cell viability was not followed by apoptotic cell death. In particular, the single exposure to atezolizumab was, in certain cells, able to promote phosphatidylserine exposure and further necrosis. Instead, radiotherapy did not promote either apoptosis or necrosis. We have previously shown that the caspases exert a key role to promote apoptotic cell death in ATC cells after the administration of sorafenib and atezolizumab [21]. So far, the cleavage of initiator caspase 8 and the executioner caspase 3 is the gold standard marker for apoptosis [23]. This study evidenced that both caspases were not cleaved after ionizing radiation thus confirming that these cells are not going to die by apoptosis.

Furthermore, this study could highlight that the ionizing radiation was able to inhibit, significantly, the ability of the ATC cells to build up colonies. Three out of four patient-derived cancer cells, the C643 cells and the normal follicular thyroid cells were characterized for a significant reduction in colony number. Except for patient 4 cells, all other cell lines showed a sensitivity similar to HNSCC cell lines [33]. This result demonstrated that ATC cells are not hypersensitive to ionizing irradiation as previously assumed by Oweida A. and colleagues [22].

The DNA repair is a fine-regulated mechanism that avoids a wrong replication of the DNA double-strand and the consequent inheritance of the disrupted encoding sequence. The first step after the DNA damage is the block of the cell cycle mediated by transcriptional regulators and genome masters such as TP53 and CDKN1A. The second step is characterized by the activation/phosphorylation of the markers of the DSB and its deputed reparatory kinases. The irradiation of ATC cells caused the over-expression of the active forms of ATM, ATR and DNA-PKcs as well as in epithelial follicular cells. Thus confirming not only that the irradiation, even at the low intensity of 4 and 6 Gy, was able to cause DSB but also that the ATC cells are able to activate DNA repair mechanisms. Nonetheless, the repair process is most probably not efficient or it must be somehow not completely working thus hampering the further cell proliferation and the ability to build up colonies.

Previous studies have highlighted that the expression of PD-L1 strictly correlates with resistance to conventional chemotherapy and, further, with the sensitivity of ATC to antibodies against PD-L1 being able to reduce tumor growth [8,9,12,34]. We have previously shown that the administration of atezolizumab in combination with multi-kinase inhibitor sorafenib or the pan-deacetylase inhibitor panobinostat could lead to ATC cell decay by promoting apoptosis and/or autophagic cell death [21]. The irradiation was able to induce the over-expression of PD-L1 in two patient-derived ATC cells. Thus, highlighting that the modulation of PD-L1 represents a potential target for personalized therapy.

## 5. Conclusions

Taken together, these results showed that the radiotherapy alone and in combination with atezolizumab was able to block the cell proliferation of ATC cells. Certainly, the loss of ability to build up colonies after irradiation would probably affect their aggressiveness and their proliferation status. Thus, supporting that these cells should undergo cell death processes. Interestingly, apoptosis was not activated as shown by the absent cleavage of the caspases 8 and 3 and by the absent exposure of phosphatidylserine.

Further research would be relevant to better identify the alternative mechanisms that could be prompted by the combined irradiation and the administration of atezolizumab in order to induce cell decay. The strong block of cell proliferation represents a relevant translational aspect for further clinical application of such treatment. Patients affected by ATC could benefit from combined ionizing therapy and immunotherapy.

## Data Availability

Not applicable.

## Supplementary Materials

The following supporting information can be downloaded at: https://www.mdpi.com/article/10.3390/life13061397/s1.
