# Supplementary figures and images for: Anti-Proliferative Effect of Radiotherapy and Implication of Immunotherapy in Anaplastic Thyroid Cancer Cells
Source: Life (Basel). 2023 Jun 15;13(6):1397. doi: 10.3390/life13061397 (PMC10301015; doi:10.3390/life13061397)

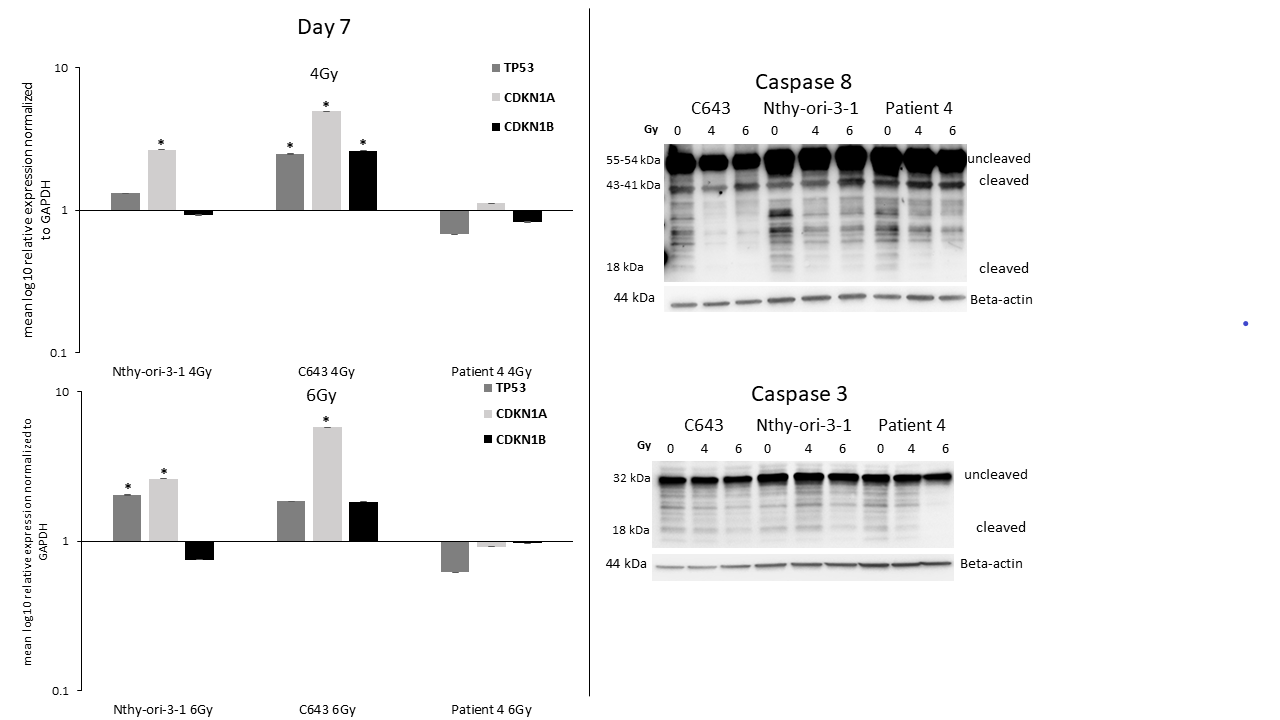

Supplement: Supplementary file 1 [file life-13-01397-s001.zip › Figure S1.tif]

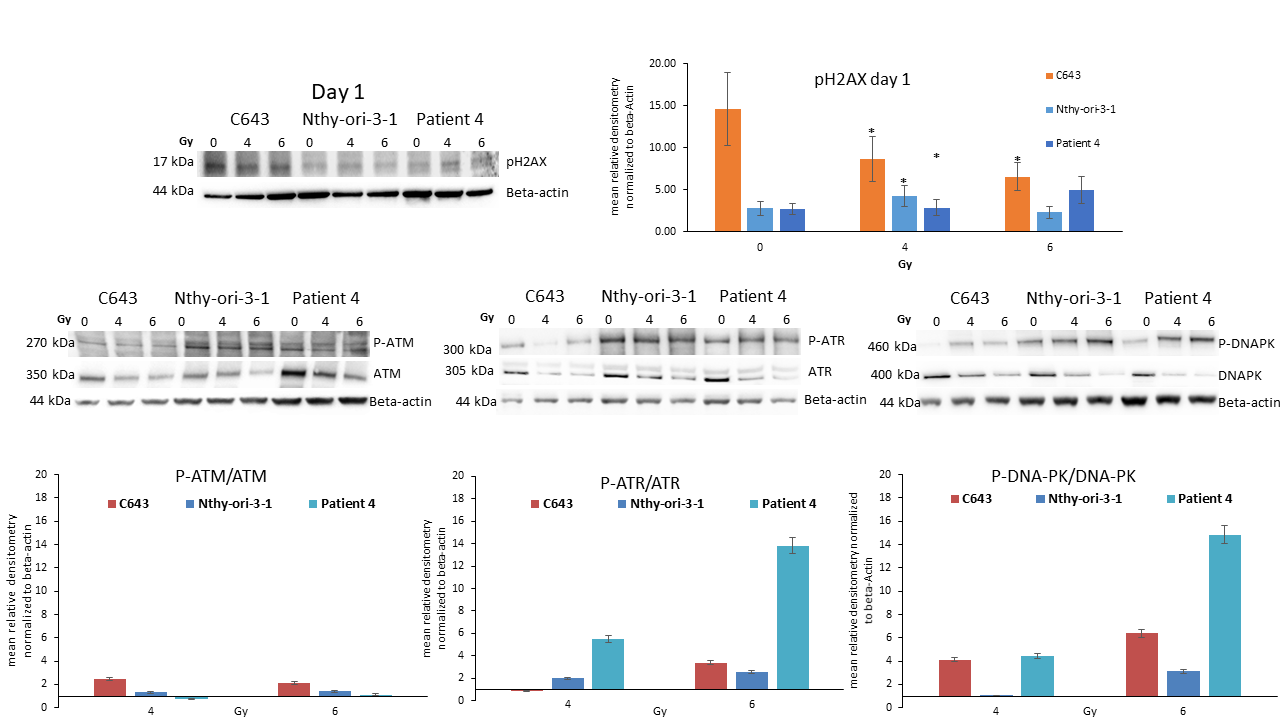

Supplement: Supplementary file 1 [file life-13-01397-s001.zip › Figure S2.tif]
